# A linguistic analysis of dehumanization toward substance use across three decades of news articles

**DOI:** 10.3389/fpubh.2023.1275975

**Published:** 2023-11-23

**Authors:** Salvatore Giorgi, Daniel Roy Sadek Habib, Douglas Bellew, Garrick Sherman, Brenda Curtis

**Affiliations:** ^1^National Institute on Drug Abuse, National Institutes of Health, Baltimore, MD, United States; ^2^Department of Computer and Information Science, University of Pennsylvania, Philadelphia, PA, United States

**Keywords:** dehumanization, substance use, addiction, New York Times, computational linguistics

## Abstract

**Introduction:**

Substances and the people who use them have been dehumanized for decades. As a result, lawmakers and healthcare providers have implemented policies that subjected millions to criminalization, incarceration, and inadequate resources to support health and wellbeing. While there have been recent shifts in public opinion on issues such as legalization, in the case of marijuana in the U.S., or addiction as a disease, dehumanization and stigma are still leading barriers for individuals seeking treatment. Integral to the narrative of “substance users” as thoughtless zombies or violent criminals is their portrayal in popular media, such as films and news.

**Methods:**

This study attempts to quantify the dehumanization of people who use substances (PWUS) across time using a large corpus of over 3 million news articles. We apply a computational linguistic framework for measuring dehumanization across three decades of New York Times articles.

**Results:**

We show that (1) levels of dehumanization remain high and (2) while marijuana has become less dehumanized over time, attitudes toward other substances such as heroin and cocaine remain stable.

**Discussion:**

This work highlights the importance of a holistic view of substance use that places all substances within the context of addiction as a disease, prioritizes the humanization of PWUS, and centers around harm reduction.

## 1 Introduction

Defined as the treatment or perception of individuals as less than human (1), dehumanization can cause harm in various contexts. Although direct consequences are often difficult to draw out, dehumanization has been shown to feed intergroup bias, abusive language, and violence (1, 2), such as in the cases of dehumanizing media campaigns against Jewish people in Nazi Germany, Rwandan Tutsis, and Arab leaders post-9/11 (3, 4). Moreover, dehumanization of patients with physical and mental health conditions has been documented extensively (1, 5–7). Most famously, people with a substance use disorder (SUD) and more broadly people who use substances (PWUS) were dehumanized throughout the War on Drugs from the 1970s to today with varying degrees of severity based on demographics and the type of drug (8). The War on Drugs, in particular, embedded dehumanizing policies into both the criminal justice and healthcare systems, dramatically increasing incarceration and negatively impacting the health and well-being of communities (9). Since the dehumanization of PWUS contributes to stigma, it is thus associated with lower support for non-discriminatory drug laws (10), inhibited help-seeking behavior (11, 12), and worse health outcomes (13). Understanding historical patterns of dehumanization and consequently discovering mitigation protocols are significant public health and public policy concerns.

Language is a key component of dehumanization. The lexicon for health problems, particularly SUDs, both reveals and affects societal responses and treatment strategies (14). Several studies have shown that dehumanizing labels induce and perpetuate explicit and implicit biases among the general public and well-trained health professionals (15–17). For instance, McGinty et al. (18) finds that stigmatizing language in American print and television news platforms about the opioid epidemic increased from 2008 to 2018, contributing to public stigma toward people with opioid use disorders. Moreover, Brown (19) argues that PWUS more effectively recognize potential harms if information is shared in a nonjudgmental way. The label of “substance abuser” conveys that the patient is the problem while “person with an SUD” conveys that the patient is not the problem but instead has a problem (17). Systematic approaches to changing the language of addiction have been spearheaded by addiction research journals and the American Society of Addiction Medicine (20, 21). While language changes more quickly in response to new information, Kelly et al. (14) shows that language evolution is slow and opts for more efficient terms, which poses barriers to adopting less dehumanizing language. Hence, it is imperative to detect, understand, and minimize dehumanizing language so people with SUDs have one less barrier to recovery (22).

Given the media's well-established role in dehumanizing social groups via sensationalist writing, Mendelsohn et al. (2) developed a computational linguistic framework for analyzing dehumanizing language. Focused on media portrayals of the LGBTQ community, their work represents the first large-scale quantitative analysis of dehumanization, allowing them to comprehensively capture media attitudes, track dehumanization over time, and capture previously untapped variations in language. Similar large-scale, multi-decade studies have measured gender and ethnic stereotypes (23), public perception of artificial intelligence (24), and the framing of immigration in political speeches (25), among others.

Based on the claim of Mendelsohn et al. (2) that the framework generalizes to other groups, we aim to apply their model to the dehumanization of PWUS. Although the dehumanization of PWUS has been well-studied, no project has quantified dehumanizing language about PWUS by American institutions across time. To do this, we use a multi-dimensional linguistic measure of dehumanization (which includes negative evaluations of a target group, denial of agency, moral disgust, and a vermin metaphor) to identify trends in dehumanization toward both PWUS (e.g., *addict* and *alcoholic*) and people who use specific substances (e.g., *marijuana* and *heroin*). This is measured across a data set of over 32 million New York Times articles from 1986 to 2020. This work contributes to our understanding of how American institutions and, in particular, mainstream media express attitudes toward marginalized populations. Furthermore, understanding these attitudes has implications for both policymakers and healthcare professionals as they respond to public health issues such as the opioid epidemic and emerging substances (26).

## 2 Related work

### 2.1 Dehumanization

Following Mendelsohn et al. (2), we use a multi-dimensional measure of dehumanization which consists of Negative Evaluation of a Target Group, Denial of Agency, Moral Disgust, and Vermin as a Dehumanizing Metaphor, which are key elements of dehumanization (1, 27). Attributing negative characteristics to dehumanized groups contributes to moral exclusion, delegitimization, and psychological distancing (28, 29). Particularly effective at distancing an outgroup is equating members of the outgroup to nonhuman entities like vermin who are portrayed as threatening, thoughtless, and emotionless (30). As such, the outgroup is perceived as undeserving of the fair rules and moral values that apply to fellow humans, which leads to abuse and violence (2, 29). A key contributor to outgroup exclusion and the negative perception of its members in many dehumanizing metaphors is moral disgust (27, 31). Groups lacking sanctity and purity are perceived as mindless and thus allowed to be hurt (32). Indeed, dehumanization involves denying agency—the ability to control one's affective, behavioral, and cognitive states—to outgroup members (1, 30). Outgroup members are thought to be incapable of rational thought or controlling their actions and are thus excluded on the basis of lacking uniquely human traits (1). While dehumanization exaggerates intergroup differences by categorically distinguishing social groups (1), it can also take subtle, involuntary, and unconscious forms (33).

### 2.2 Media portrayals of people who use substances

Previous work has brought attention to how American media coverage of individuals who use drugs has historically been and continues to be dehumanizing (34). Mass media partakes in what Reinarman and Duskin (35) call, “the routinization of caricature—rhetorically recrafting worst cases into typical cases, and profoundly distorting the nature of drug problems in the interest of dramatic stories.” Durham et al. (36) showed how newspapers and television historically paint misleading crime images. Similarly, Coomber et al. (37) showed that drug-related stories are particularly fraught with stereotypical images to increase viewership. For instance, Boyd et al. (38) explained how drug traffickers in movies assume the role of an out-of-control “outsider” who “threatens the world order of white, middle-class protestant morality.” Young et al. (39) argue that certain people, such as police officers, are particularly susceptible to believing in media stereotypes. Media influences public opinion and vice versa (40), and as Gentzkow and Shapiro (41) states, “news content has a powerful impact on politics, with ideologically diverse content producing socially desirable outcomes”. More than misleading, Murji (42) argues that the media can do harm by instigating drug crackdowns.

### 2.3 Related computational work

Computational linguistics and natural language processing methods have been used in several substance use related tasks. These include public perceptions of medical cannabis use (43), Reddit-based self-reported barriers to treatment seeking (12), and identifying emerging drug-related words and slang (44). At the population level (e.g., U.S. counties and states), a handful of studies have examined social media language and substance use rates, such as excessive drinking (45, 46), opioid mortality (47, 48), and, more generally, pharmacovigilance (49). Similar to dehumanization, there is a growing body of work focused on computational work identifying stigma toward people who use substances (50–52).

## 3 Data

### 3.1 New York Times corpus

We use a corpus of 3.05 million New York Times articles spanning from 1986 to 2015, first collected by Fast and Horvitz (24) and used by Mendelsohn et al. (2) to assess dehumanization of LGBTQ people. We further supplement this data set with 229,235 more recent articles from 2016 to 2020. To collect this additional data, we first repeatedly queried the New York Times Archive API^1^ to list the metadata of all articles published for the years 2016–2020. This metadata included the URL of each article, which we used to download each article's full text. We then scraped the contents of these webpages using the Beautiful Soup Python package.^2^ Following Mendelsohn et al. (2), we retained articles related to news such as those coming from the World, Politics, Sports, Opinions, and Health sections and removed articles from the Arts and Movies sections as these are not typically news-related.

The final data set thus spanned from January 1986 to December 2020 and included 3.28 million articles, containing 39.6 million paragraphs, which can be further broken down into 96.9 million sentences. All articles are date stamped to allow for time-based collation.

### 3.2 Substance use keywords

**Figure 1:**
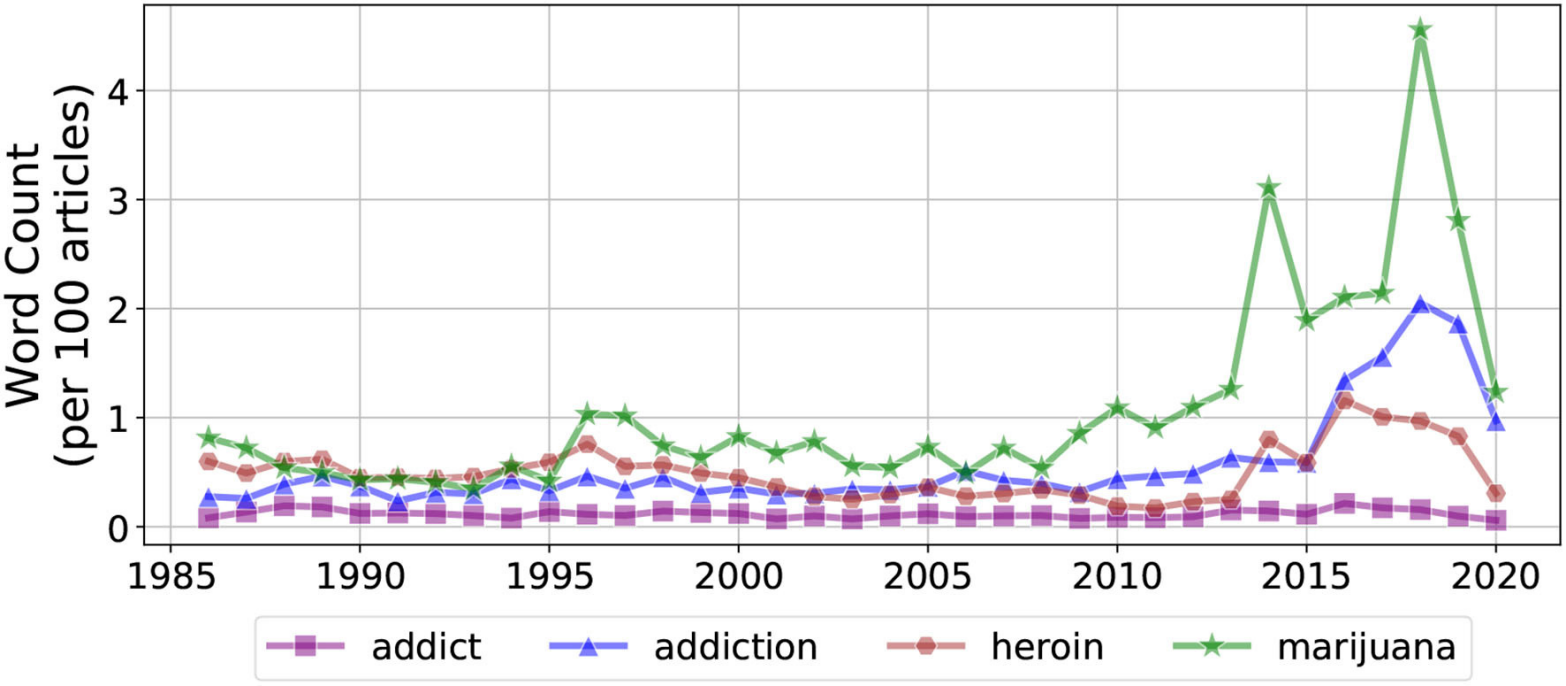
Keyword counts, per 100 articles, over time for *addict, addiction, heroin*, and *marijuana*.

In order to identify New York Times articles about substance use, we consider two classes of keywords: (1) keywords associated with PWUS and (2) keywords representing substances themselves, which we assume is a proxy for people using that specific substance. For the first class, we consider *addict(s), addiction, alcoholic(s)*, and *alcoholism*.^3^ For the second class, we consider *cocaine, heroin, marijuana, methamphetamine(s)* , *opioid(s), opiate(s), oxycontin, percocet*, and *xanax*. Figure 1 shows the frequency of several keywords.^4^ Due to their low frequency, we excluded *methamphetamine(s)* , *opioid(s), opiate(s), oxycontin, percocet*, and *xanax* from further analysis. In particular, the terms *opioid(s)* did not become popular until roughly 2010, while the other terms (*oxycontin, percocet*, and *xanax*) remained rare across all decades.

Since the goal of the study is to understand attitudes toward people who use substances, a natural comparison group would be people who do *not* use substances. This comparison is done to contrast both overall levels of dehumanization and compare trends over time. Unfortunately, due to the keyword approach, there is no way to accurately identify this group as there are no common single words or phrases which refer to people who do not use substances. Thus, following Mendelsohn et al. (2), we compare the substance use keywords to the keyword *american(s)*. Results from Mendelsohn et al. (2) showed that there was little change in dehumanization toward *american(s)* across time and, therefore, we will use these keywords to compare overall levels of dehumanization to the substance use keywords.

## 4 Methods

Here we use the computational framework outlined by Mendelsohn et al. (2) and, unless otherwise stated, use their recommended algorithmic settings.

### 4.1 Word embeddings across time

Several of the methods developed by Mendelsohn et al. (2) rely on word embeddings (i.e., vector representations of words) to measure their semantic change across time. As such, we begin by examining the words closest in embedding (or semantic) space to our substance use keywords and how these neighbors change over time.

We begin by training a word embedding model over the entire New York Times corpus, using the word2vec skip-gram model (53). This is done via the Gensim software package (54) using all default parameters except that models are trained for five iterations and with a window size of 10 words. The resulting word embeddings are then used to initialize subsequent word2vec models trained for each year of the data (i.e., 35 separate word2vec models). This process is repeated 10 times and, for each year, results are averaged over the 10 models, in order to smooth out any randomness in each training.

Substance use keyword vectors are then created using a weighted average of all forms of the keyword. For example, vectors for *addict* and *addicts* are combined into a single vector by weighting individual vectors by their frequency. We then find each substance use keyword's closest neighbors in the yearly embedding space by computing the cosine distance between the keyword vector and all other vectors in the embedding space. We report the 10 words with the smallest average distance.

### 4.2 Negative evaluation of target group

Negative evaluation of a target group is operationalized via three measures: paragraph-level valence, word embedding valence, and connotation frames of perspective. These methods are applied at the paragraph, word, and sentence level, respectively, in order to measure dehumanization across different lengths of context. Each measure is applied to yearly segments of the corpus from 1986 to 2020.

#### 4.2.1 Paragraph-level valence

Valence is measured using the valence dimension of the NRC Valence, Arousal, and Dominance (VAD) lexicon (55). This lexicon contains 20,000 words with valence scores ranging from 0 (most negative valence) to 1 (most positive valence). The negative end of the lexicon contains words such as “shit,” “nightmare,” and “toxic,” while the positive end contains words such as “enjoyable,” “generous,” and “happy.” We then calculate the average valence of each paragraph, considering only paragraphs which contain a substance use keyword. Paragraphs are used as the unit of analysis in order to give more context to the substance use keywords (as opposed to sentences, for example).

#### 4.2.2 Word embedding valence

This method measures valence at the word level by looking at the valence of the nearest neighbors to each substance use keyword. Similar to the methods outlined in Section 4.1, we identify the 500 closest words to each keyword by finding the minimal cosine distance across all words. We then assign each neighbor a valence score using the valence dimension of the NRC VAD lexicon, taking the average valence score across all 500 neighbors.

#### 4.2.3 Connotation frames of perspective

This method enables us to measure directed sentiment through a lexicon of 900 English verbs (56). Each verb is weighted to represent the writer's perspective toward the verb's subject and object. For example, the verb *harm* has a negative weight (−0.87) toward the subject (i.e., the person doing the harm) and a positive weight (0.20) toward the object (i.e., the receiver of the harm). We extract all subject-verb-object tuples containing at least one of the substance use keywords. This is done using spaCy's dependency parser.^5^ We then use the lexicon to measure the writer's perspective toward the keyword and average the verb weights over all tuples.

### 4.3 Denial of agency

Denial of Agency is operationalized via two measures: connotation frames and word embedding dominance. These measure Denial of Agency at both the sentence and word level, respectively.

#### 4.3.1 Connotation frames of agency

Here we use the same methods outlined in Section 4.2.3 but use a lexicon designed to measure agency (57). Similar to the Connotation Frames of Perspective, this lexicon consists of verbs used to measure agency between a subject and an object. Words such as “harm” and “fires” are labeled as high agency for the subject, whereas words such as “relishes” and “inherits” represent low agency. This lexicon uses binary scores for each verb, whereas the Connotation Frames of Perspective contained real-valued scores. Since we are interested in the agency of the individual using the substance, we only consider subject-verb-object tuples where the subject is a substance use keyword. Therefore, we calculate the fraction of subject-verb-object tuples where the subject has high agency.

#### 4.3.2 Word embedding dominance

We use the dominance dimension from the NRC VAD lexicon (55). This lexicon represents dominance via 20,000 English words, which are each weighted between 0 and 1. The highest-weighted words in the lexicon (representing high dominance) are “power,” “leadership,” and “success,” while the lowest-weighted words (representing low dominance) are “weak,” “frail,” and “empty.” To measure dominance, we use the same approach as outlined in Section 4.2.2. Specifically, we compute the average dominance of the 500 nearest neighbors for each substance use keyword.

### 4.4 Moral disgust

To measure Moral Disgust, we use the sanctity/purity dimension of the Moral Foundations lexicon (58), taking the negative (or vice) end of this dimension. This dimension contains 46 words such as “disgust*,” “gross,” and “wretched*” (where an asterisk will match any word that, for example, begins with “disgust” such as “disgusting”). Unlike the NRC VAD lexicon, the words in the Moral Foundations lexicon are not weighted. Using this lexicon, we create a moral disgust vector by taking the average word2vec embedding of all words within the negative sanctity/purity dimension, weighting each embedding by the word's frequency. This is done for each year, resulting in 35 moral disgust vectors. We then calculate the yearly semantic similarity between the moral disgust vectors and each substance use keyword vector using a cosine similarity metric. A larger cosine similarity represents higher semantic similarity.

### 4.5 Vermin as a dehumanizing metaphor

The association between substance use and vermin is measured by calculating the semantic similarity between the substance use keywords and vermin. First, we create a vermin vector by taking the average word2vec embedding for “bedbug(s),” “cockroach(es),” “fleas,” “rat(s),” “rodent(s),” termite(s),” and “vermin,” where each vector is weighted by its frequency. Then, for each year, we calculate the cosine similarity between the vermin vector and the substance use keyword vectors, where a larger value represents higher semantic similarity.

### 4.6 Error analysis

We end with two qualitative error analyses. First, we manually inspect the paragraphs with the highest and lowest valence scores to see if our methods correctly identify dehumanization. To do this, we identify paragraphs with the highest (≥0.7; i.e., lower dehumanization) and lowest ( ≤ 0.3; i.e., higher dehumanization) normalized valence scores from the NRC Valence lexicon (on a scale from 0 to 1). Valence is the average valence of the paragraph, and all paragraphs have a minimum of 15 words in order to provide a larger context to the keywords. To determine if the valence scores match the direction of dehumanization, we annotated each paragraph as being correctly labeled by the lexicon. In other words, we determined if each paragraph showed decreased dehumanization for high valence scores and increased dehumanization for low valence scores. Three authors separately annotated each paragraph as being correctly (valence score correctly matches the level of dehumanization) or incorrectly (valence score incorrectly matches the level of dehumanization) labeled by the lexicon, or not relevant (NR) when the paragraph is not referring to substances or substance use. The final labels of correct, incorrect, and NR were assigned by a majority vote across the three annotations.

Second, we note that the NRC Valence lexicon contains all of the keywords used in this study and several related words which reference substance use (e.g., *methadone*, *lsd*, and *amphetamines*). In particular, these words were all negatively valenced, and therefore any conversation around substance use will tend toward more dehumanization when measured with the NRC lexicon. Therefore, we measure paragraph-level sentiment using the positive emotions category in the 2015 version of the Linguistic Inquiry and Word Count (LIWC) dictionary, since this category does not contain substance-related words (59). LIWC is a manually curated dictionary that measures constructs such as psychological processes (e.g., anxiety and sadness) and linguistic dimensions (e.g., pronouns and verbs). To measure paragraph-level sentiment using LIWC we count the number of words within each paragraph that are in LIWC's positive emotions category and divide the count by the total number of words in the paragraph.

## 5 Results

### 5.1 Word embeddings across time

Table 1 shows the most similar words (or nearest neighbors) as measured by the (minimum) cosine distance between the word2vec representations of the keywords *addict* and *addiction* and all other words in the embedding space. In 1986, we see words associated with specific substances (“heroin”), connections to *alcoholism*, “abuse” and “abusers”, as well as mental (“psychosis” and “schizophrenia”) and eating disorders (“bulimia”). In 2000, we see further mentions of eating disorders (“anorexia” and “bulimia”) as well as “obesity”, words related to sex (“venereal” and “cybersex”). This continues in 2010, with “hypersexuality”/ “prostitutes” and “bulimia”/“bulimic”. Finally, in 2020 we see a larger number of words related to mental disorders: “bipolar,” “schizophrenia,” and “adhd.” Notably, “abuser” drops out of the top 10 results in 2020. Across all years, we see “heroin” closely related to *addict*.

**Table 1:** Addict and addiction: nearest neighbors in embedding space.

**1986**		**2000**		**2010**		**2020**	
**Addict**	**Addiction**	**Addict**	**Addiction**	**Addict**	**Addiction**	**Addict**	**Addiction**
Abuser(s)	Alcoholism	Heroin	Alcoholism	Heroin	Alcoholism	Heroin	Alcoholism
Alcoholics	Abuse	Cybersex	Bulimia	Abuser	Opi(ate/oid)	Opiate	Bipolar
Intravenous	Drug(s/-)	Abuser	Obesity	Addled	Venereal	Womanizer	Schizophrenia
Drug(s/-)	Schizophrenia	Addled	Compulsivity	Opiate	Psychosis	Alcoholism	Psychosis
Methadone	Bulimia	Opiate(s)	Venereal	Prostitutes	Drug(s/-)	Addled	ADHD
Heroin	Psychosis	Adolescents	Alcohol	Bulimic	Bulimia	Methadone	Homelessness
Users	Opiate(s)	Drug	Cybersex	Hookers	Anorexia	Dope	Anorexia
Teenagers	Diabetes	Compulsive	Anorexia	Methadone	Hypersexuality	Bipolar	Opi(ate/oid)
Opiates	Cocaine	GHB	Drug	Psychotic	Hypoactive	Prostitute	Bulimia
Hemophiliacs	Venereal	LSD	Opiate(s)	Vicodin	Heroin	Compulsive	Depression

Similar words are collapsed (e.g., opiate and opioids) if they are both found in the top 10 results. (-) indicates words which contain hyphens which are split by the tokenizer (e.g., drug-abuse).

In Table 2, we compare *marijuana* to *heroin*. Again, in 1986 the most similar words are other substances: “cocaine,” “heroin,” “marijuana,” and “hashish.” We also see words related to legality: “illicit” for both *marijuana* and *heroin* and “trafficking” for *heroin*. As time progresses, starting in 2010, *marijuana* becomes more closely related to legality (“decriminalizing” and “legalizing”), “dispensaries” (i.e., places to legally purchase marijuana), and other legal substances, such as “alcohol,” “nicotine,” and “tobacco.” *Marijuana* is also still closely related to “cocaine” and “methamphetamine(s)”, neither of which are legal. *Heroin* continues to be closely related to other substances as time progresses. Across all 4 years, “cocaine” remains the most related word. Other substances such as “methamphetamine(s),” “vicodin,” and “opiates” appear in 2000. In 2010, we see “oxycodone” and “oxycontin”, both of which are prescription painkillers. Finally, in 2020, we see “overdose” and “fentanyl”, a synthetic opioid.

**Table 2:** Marijuana vs. heroin: nearest neighbors in embedding space.

**Marijuana**				**Heroin**			
**1986**	**2000**	**2010**	**2020**	**1986**	**2000**	**2010**	**2020**
Cocaine	Amphet.	Dispensaries	Cannabis	Cocaine	Cocaine	Cocaine	Cocaine
Heroin	Smokeless	Cannabis	Amphet.	Marijuana	Amphet.	Hashish	Amphet.
Hashish	Cannabis	Alcohol	Decriminalizing	Trafficking	Hashish	Amphet.	Fentanyl
Opium	Alcohol	Cocaine	Legalizing	Hashish	Opi(um/ates/oids)	Oxyco(ntin/done)	Overdose
Drug(s)	Hashish	Amphet.	Cocaine	Drug(s)	Addicts	Drugs	Opiates
Amphet.	Cocaine	Tobacco	Dispensaries	Narcotics	Vicodin	Vicodin	Oxycodone
PCP	Ketamine	Firearms	Hashish	Opiates	Dilaudid	Barbiturates	Hashish
Narcotics	Cigarettes	Decriminalizing	Tobacco	Smokable	Ecstasy	Opiates	Ketamine
Illicit	LSD	Dispensary	Nicotine	Illicit	Ketamine	Addicts	Painkillers
Alcohol	Unapproved	Hashish	Cigarettes	Addict(s/ion)	Hydromorphone	Percocet	Vicodin

Similar words are collapsed (e.g., oxycontin and oxycodone) if they are both found in the top 10 results. amphet. = (meth)amphetamine(s).

### 5.2 Components of dehumanization

**Figure 2:**
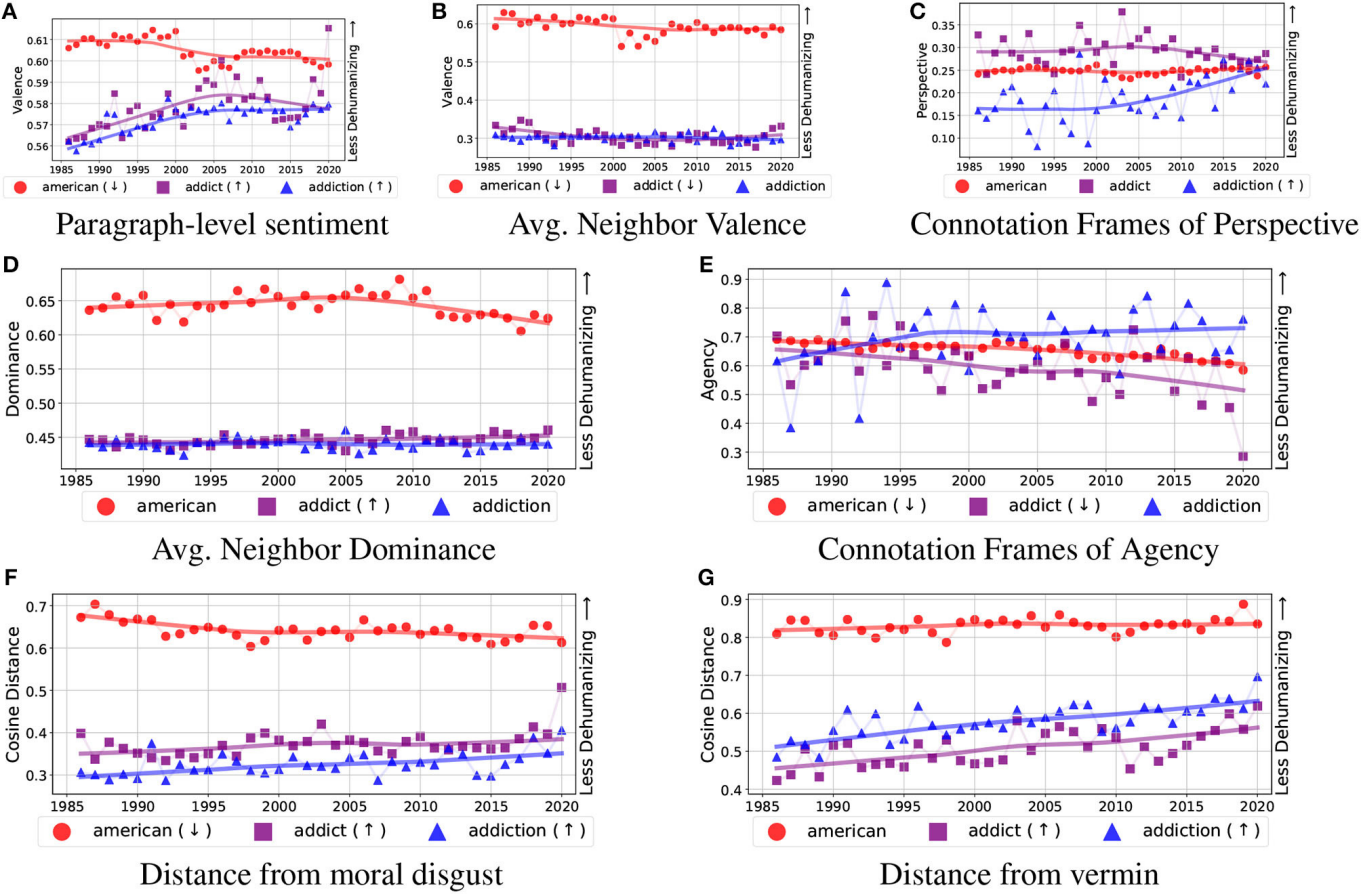
Comparison of *addiction* (blue triangles) and *addict* (purple squares) to the control keyword *american* (red circles) across all four measures of dehumanization: negative evaluation of a target group **(A–C)**, denial of agency **(D, E)**, moral disgust **(F)**, and vermin metaphor **(G)**. A significant increase or decrease over time is indicated with ↑ and ↓, respectively, next to the label in the legend in each subplot. In all plots, smaller values on the vertical axis represent *increased* dehumanization.

In Figure 2, we see the temporal trends in the linguistic dehumanization measures for the *addict* and *addiction* keywords as well as the control keyword *american*. Note that smaller values on the vertical axis represent more dehumanization, while larger values represent less dehumanization. Across five of the seven measures, we find that *american* has larger values than both *addict* and *addiction*, suggesting increased dehumanization in the language discussing PWUS and matching results from Mendelsohn et al. For both connotation-frame measures (agency and perspective), we see all three keywords close to each other when compared to the remaining measures.

**Figure 3:**
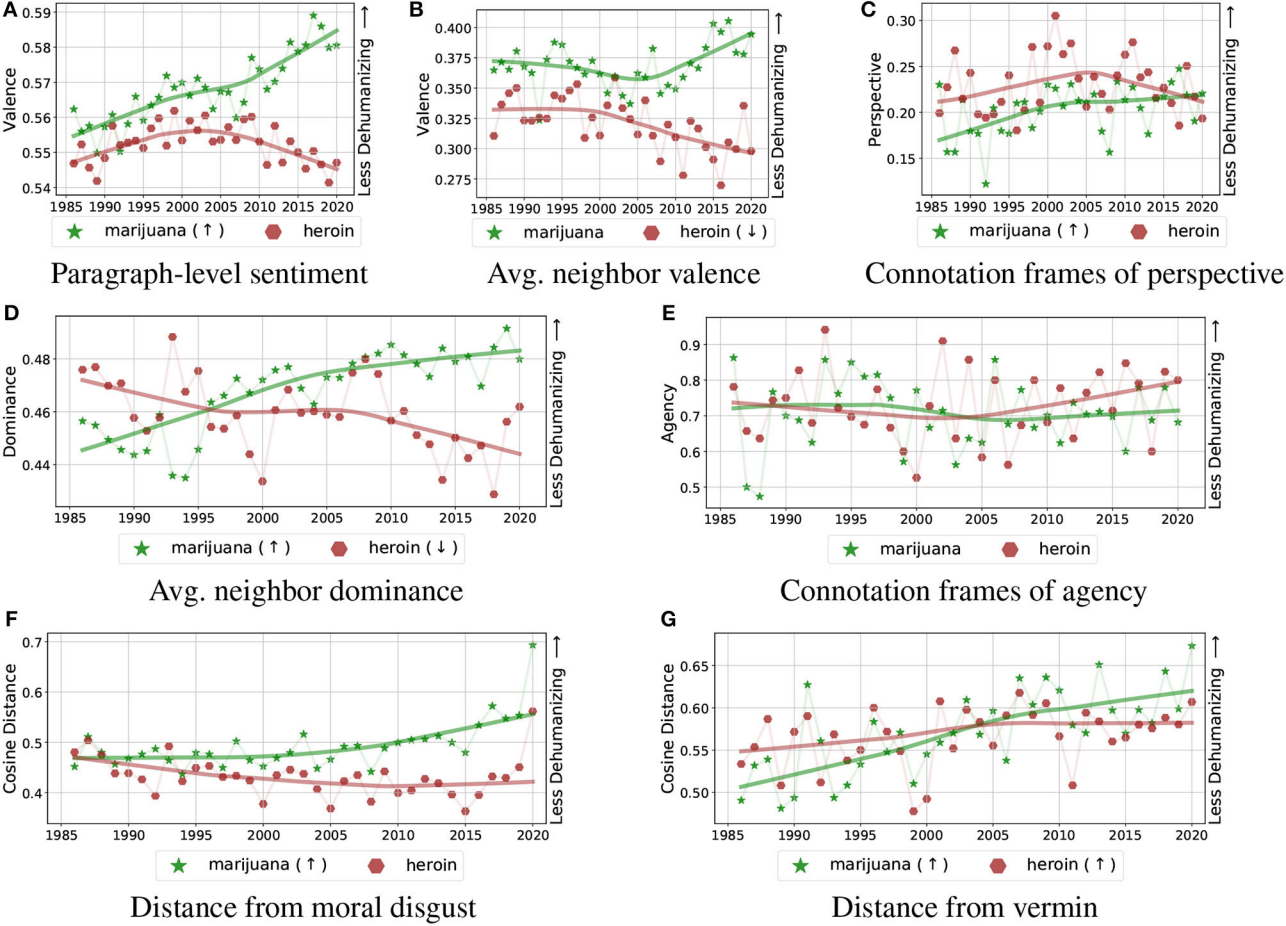
Comparison of *marijuna* (green stars) and *heroin* (brown hexagons) across all four measures of dehumanization: negative evaluation of a target group **(A–C)**, denial of agency **(D, E)**, moral disgust **(F)**, and vermin metaphor **(G)**. A significant increase or decrease over time is indicated with ↑ and ↓, respectively, next to the label in the legend in each subplot. In all plots, smaller values on the vertical axis represent *increased* dehumanization.

Figure 3 compares *marijuana* to *heroin*. Across four (out of seven) measures, we see the two keywords diverge: (a) paragraph-level sentiment, (b) average neighbor valence, (d) average neighbor dominance, and (f) distance from moral disgust. In each of these four plots, we see, over time, more humanization in language surrounding *marijuana* and more dehumanization in the language around *heroin*. Similar to the results we saw in Figures 2C, E, the connotation-frames measures show both keywords as having relatively similar values of linguistic dehumanization across time.

Turning to the components of dehumanization, we see: (1) all four keywords moving away from vermin, and (2) three out of four keywords becoming more distanced from disgust (*heroin* has no change). Thus, consistently across both Moral Disgust and Vermin Metaphor, we see more linguistic humanization regardless of the substance keyword being examined. Across the two Denial of Agency measures, we see mixed results, which depend on both the measure and the keyword: (1) *addiction* shows no change, (2) *addict* goes up in one measure (average dominance) and down in another (connotation frames of agency), (3) *marijuana* increases in one measure (average dominance) and shows no change in another (connotation frames of agency), and (4) *heroin* decreases in one measure (average dominance) and shows no change in another (connotation frames of agency). Similarly, Negative Evaluation of a Target Group shows mixed results for some keywords. Both *addiction* and *marijuana* increase for two of the three measures, while no change is seen for the third measure. The keyword *addict* differs across all three measures, while *heroin* shows no change in two out of the three measures (and decreases on the third measure).

#### 5.2.1 Summary

Since all of the proposed measures are proxies for some component of dehumanization (e.g., distance from the vermin embedding is a proxy for vermin metaphors) and no single component is proof of humanization/dehumanization, we report summaries across each keyword. These results are summarized in Table 3. For each keyword, we summarize the temporal trend for each measure of dehumanization. Blue up arrows indicate an increase in *humanization* (i.e., a positive product-moment correlation, significant at *p* < 0.05), whereas red down arrows indicate an increase in *dehumanization* (i.e., a negative product-moment correlation, significant at *p* < 0.05). We then add the number of significant results, where up arrows (humanization) are counted as positive 1 and down arrows (dehumanization) are counted as −1 (thus, totals can range from −7 to 7). The resulting total provides a measure for the strength of the change in dehumanization over time. Results show that people who use marijuana, in particular, have become less dehumanized over time, with an increase in linguistic humanization across five out of seven measures. Articles using general terms for PWUS (*addict, addiction*, and *alcohol* keywords) saw slight increases in linguistic humanization (increases in two measures each). Discussions around the remaining keywords saw no change (*alcoholism*), small increases (*alcoholic*), or small decreases (*cocaine* and *heroin*) in linguistic humanization.

**Table 3:** Summary table for all substance related keywords.

	**Negative evaluation of target group**			**Denial of agency**		**Moral disgust**	**Vermin metaphor**	**Total**
	**Paragraph- level sentiment**	**Connotation frames of perspective**	**Average neighbor valence**	**Connotation frames of agency**	**Average neighbor dominance**	**Similarity to disgust**	**Similarity to vermin**	
Addict	↑	–	↓	↓	↑	↑	↑	+2
Addiction	↑	↑	↓	↓	–	↑	↑	+2
Alcoholic	↑	–	–	–	–	–	–	+1
Alcoholism	–	–	↓	–	↓	↑	↑	0
Alcohol	↑	–	↑	–	↓	↑	–	+2
Cocaine	↓	–	↑	–	↓	–	–	−1
Heroin	–	–	↓	–	↓	–	↑	−1
Marijuana	↑	↑	–	–	↑	↑	↑	+5

↑ significant increase over time (positive product-moment correlation, *p* < 0.05), ↓ significant decrease over time (negative product-moment correlation, *p* < 0.05), – no change (product-moment correlation not significant). Total column is the sum of all significant results, operationalized as +1 for significant increases, −1 for significant decreases, and 0 for no change. Thus, higher magnitude positive totals show more humanization over time (on average), more negative totals show more dehumanization over time (on average), and 0 shows no change (on average).

### 5.3 Error analysis

In Table 4 (*addict* and *addiction*), Table 5 (*marijuana*), and Table 6 (*heroin*), we show the top- and bottom-most valenced paragraphs in the data set. Words within the example paragraphs are highlighted red (negative) when the word has a valence score of ≤ 0.3 and highlighted blue when the word has a valence score of ≥0.7 in the NRC Valence lexicon. The final labels consist of 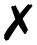 (valence score correctly matches the level of dehumanization), 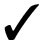 (valence score incorrectly matches the level of dehumanization), and NR (not relevant).

**Table 4:** Addict and addiction paragraphs with highest (top five rows) and lowest (bottom five rows) valence, as measured by the NRC Valence lexicon.

**Valence**	**Text**	**Year**	**Correct**
0.77	His knowledge of both young dealers and **addiction** has transformed him into the ideal counselor for the program.	1992	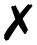
0.76	Mr. Jorgens views his **addiction** in a more spiritual light. Skating “provides the clarity to make you feel good about yourself, to feel at one with yourself, to feel a sense of meditation each time you do it”	2001	NR
0.76	From one swimming **addict** to another, I thank Ms. Tsui profusely for her illuminating insights on “the magic of water.” I also miss swimming most of all.	2020	NR
0.75	Most **addicts** acknowledge that recovery is a day-to-day journey. What Taylor at 50 can offer are life lessons.	2009	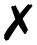
0.74	Whether or not the **addict** ever gets well, Mr. Moyers said, “families have to take care of themselves. They can't let the **addict** walk over their lives”.	2013	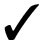
0.34	Shame and stigma are the exact opposite of what fights **addiction**. If shame worked, so would criminal penalties for drug use, which haven't exactly ended **addiction**.	2016	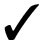
0.33	Conversely, 60% of people with a substance abuse disorder also suffer from another form of mental illness. Still, it's unclear whether **addiction** predisposes someone to mental illness, or vice versa.	2011	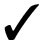
0.32	Studies also suggest that long-term steroid abusers suffer psychiatric disturbances similar to those of cocaine **addicts**, including impaired judgment, increased irritability, anxiety, panic and paranoid delusions.	1989	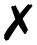
0.30	Drugs. You remember—drugs, as in drug **addiction**, drug crime, drug disease, drug homeless, drug madness, drug guns, drug blood and drug babies. And as in The War Against Drugs, declared by Washington, way back, about three years ago.	1992	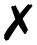
0.30	OTTAWA—A loner. A drug **addict**. A criminal. A drifter. And lately, an Islamic radical.	2014	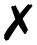

Keywords (*addict* and *addiction*) are highlighted in black, high valence words in blue (valence scores ≥0.7), and low valence words in red (valence scores ≤ 0.3). In the Correct column: 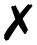 the paragraph was correctly labeled as positively (or negatively) valenced, 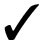 the paragraph was incorrectly labeled as positively (or negatively) valenced, and NR (not relevant) means the paragraph does not refer to substance use or PWUS. All examples have a minimum of 15 words, in order to provide enough context.

**Table 5:** Marijuana paragraphs with highest (top five rows) and lowest (bottom five rows) valence, as measured by the NRC Valence lexicon.

**Valence**	**Text**	**Year**	**Correct**
0.79	After traveling for a while in Asia, however, he has dedicated his efforts to promoting **marijuana** and its culture	2005	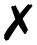
0.75	He knows that it earns money dealing **marijuana** and methamphetamine, but that the income is not enough for several members, who have legitimate day jobs.	2007	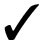
0.74	Today, his gold medal for snowboarding's giant slalom was taken away because he had tested positive for **marijuana**, Francois Carrard, director general of the International Olympic Committee, said.	1998	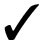
0.74	As for what was really going on at Brooklyn Farms, Veksler is emphatic : “Honest to God, I've never grown **marijuana** in my life. But now, knowing what I know, I could be a master farmer”.	2013	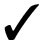
0.73	The ballot proposal to allow the social use of **marijuana** at some bars or nightclubs drew passionate responses across the city. Some restaurant owners and event planners said they would love to host marijuana-friendly dinner parties or galas, but the Colorado Restaurant Association is “adamantly against” the idea, said a spokeswoman for the group, Carolyn Livingston.	2015	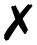
0.37	Still, does this cannabinoid mutation simply correlate with less anxiety, and less addiction to **marijuana**—or does it cause them?	2015	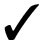
0.36	Mr. Pitera, whose gang sold cocaine, heroin and **marijuana**, was convicted of committing six murders in his racketeering and drug operations. Mr. Pitera and his followers dismembered their victims—drug dealers, addicts and murderers—and buried the remains in a wooded section of Staten Island.	1992	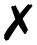
0.36	They include eliminating federal incarceration for drug possession and reducing sentences for other drug offenses; legalizing **marijuana** at the federal level; limiting solitary confinement; and abolishing the death penalty and mandatory minimum sentencing.	2019	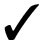
0.35	The program allows medical **marijuana** for certified patients who have cancer, H.I.V. / AIDS, Parkinson's disease, multiple sclerosis, intractable spasticity caused by damage to the nervous tissue of the spinal cord, epilepsy, inflammatory bowel disease, neuropathies and Huntington's disease.	2016	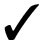
0.34	Drug abuse is American's No. 1 health problem. The abuse of alcohol heroin, cocaine and crack, **marijuana**, PCP (angel dust), LSD, stimulants and sedatives causes deaths, severe medical and psychiatric problems and disabilities.	1987	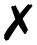

Keyword (*marijuana*) is highlighted in black, high valence words in blue (valence scores ≥0.7), and low valence words in red (valence scores ≤ 0.3). In the Correct column: 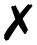 the paragraph was correctly labeled as positively (or negatively) valenced, 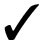 the paragraph was incorrectly labeled as positively (or negatively) valenced, and NR (not relevant) means the paragraph does not refer to substance use or PWUS. All examples have a minimum of 15 words, in order to provide enough context.

**Table 6:** Heroin paragraphs with highest (top five rows) and lowest (bottom five rows) valence, as measured by the NRC Valence lexicon.

**Valence**	**Text**	**Year**	**Correct**
0.76	The biggest “seductress” in his life was **heroin**, he writes, which he relied on to anesthetize him from the “blah blah blah” of show business, something he did not enjoy as much as Jagger.	2010	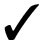
0.74	Giving to charity, paying taxes, and receiving information about future events all activate the same neural pleasure circuit that's engaged by **heroin** or orgasm or fatty foods.” (David Linden, “The Compass of Pleasure”)	2011	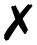
0.72	As a **heroin** dealer in Rhode Island, Jose Vasquez made $2,000 a day. He said he had a way with his customers. He took his best clients out for dinner and bought them presents on their birthdays.	2012	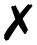
0.72	In 1986, when Drew was born, crack was thriving on Dodworth, which had already had a good, long run with **heroin**. Tata was young, swamped by too many children—after Drew, she gave birth to a daughter. Tata and Drew were both growing up, and he had lots of physical energy, just as she did.	2002	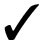
0.71	Though he knew the stories of renowned musicians like Charlie Parker or Chet Baker who used **heroin**, he said he was never drawn to it for the romance. “It's more like the thing itself,” he said. “Honestly, I don't think anybody I know romanticized it as much as they liked it. It's got good qualities.	2014	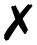
0.34	The tragedy of Patricia Marback's **heroin** overdose death (news article, Aug. 14) says more about the hazards of our drug policies than about the dangers of the drug itself.	1995	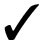
0.33	This gang deals in murder, guns and narcotics, including marijuana, cocaine and **heroin**,” she said.	1986	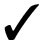
0.33	Some clinics have used acupuncture to fight chronic pain or the agonies of withdrawal from addiction to **heroin**, alcohol and, most recently, cocaine.	1986	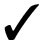
0.32	Mr. Tavarez pleaded not guilty after his arrest last May; at the time of his arrest he was suspended without pay. On Monday, he pleaded guilty to three charges: robbery conspiracy; conspiracy to distribute **heroin** and cocaine; and the use of a firearm in the course of those crimes.	2011	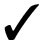
0.29	Many prescription overdose deaths and most **heroin** overdose deaths are in combination with another sedative, usually alcohol. That makes these terrible accidents all the more preventable.	2015	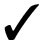

Keyword (*heroin*) is highlighted in black, high valence words in blue (valence scores ≥0.7), and low valence words in red (valence scores ≤ 0.3). In the Correct column: 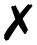 the paragraph was correctly labeled as positively (or negatively) valenced, 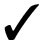 the paragraph was incorrectly labeled as positively (or negatively) valenced, and NR (not relevant) means the paragraph does not refer to substance use or PWUS. All examples have a minimum of 15 words, in order to provide enough context.

Across all keywords, we see examples of the valence lexicon correctly and incorrectly identifying linguistic dehumanizing content. Several examples where linguistic dehumanization is incorrectly identified are a result of the following categories of words being negatively valenced: (1) substances and substance use (“drug” and “withdrawal”), (2) criminal justice (“arrest,” “incarceration,” and “sentencing”), and (3) mental and physical health (“pain,” “illness,” and “anxiety”). For *addict* and *addiction* in Table 4, we see that other types of addiction (skating and swimming) are being classified as related to substance use due to the ambiguity in the keyword approach. These two examples are thus labeled as not relevant (NR).

Table 7 compares the paragraph-level sentiment analysis using both the NRC Valence lexicon and LIWC. Here we see that the two sentiment measures agree on six out of eight keywords. The measures disagree on *addict*, where NRC shows an increase in valence and LIWC exhibits no change, and *cocaine*, where NRC shows a decrease in valence and LIWC increases.

**Table 7:** Comparison of lexical-based methods.

	**NRC valence**	**LIWC positive emotions**	**Agree**
Addict	↑	–	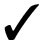
Addiction	↑	↑	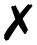
Alcoholic	↑	↑	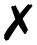
Alcoholism	–	–	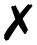
Alcohol	↑	↑	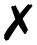
Cocaine	↓	↑	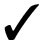
Heroin	–	–	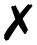
Marijuana	↑	↑	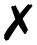

The NRC Valence lexicon contains negatively valenced substance use keywords, whereas LIWC does not. ↑ indicates a significant increase over time (positive product-moment correlation, *p* < 0.05), whereas ↓ indicates a significant decrease over time (negative product-moment correlation, *p* < 0.05), with – representing no change (product-moment correlation not significant). The Agree column indicates if the two lexica agree (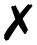) or disagree (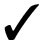) in the direction of the measured dehumanization.

Taken together, while the NRC lexicon by default drives results toward dehumanization and thus causes misclassifications, overall trends hold when using an alternative lexicon (LIWC) that does not have the same limitations.

## 6 Conclusions

America's War on Drugs extended to dehumanizing the people who use them. Our findings suggest that PWUS have been dehumanized in popular media for decades. Overall levels of dehumanization remain high when compared to baselines (e.g., *americans*). While temporal trends suggest that conversations have been shifting since the 1980s (i.e., toward decriminalizing and systemic issues such as mental health), there seem to be differences across substances. Marijuana, in particular, has become less dehumanized over time. These trends dovetail with annual polls showing increased support for legalizing marijuana (60). On the other hand, substances such as cocaine and heroin show little change.

While the results show that people who use marijuana have become less dehumanized over time, we note that there a long history of stigmatization and dehumanization toward this group. This dates back to the 1930s where it was connected to violent crimes and immigration. This switched contexts in the 1960s to dehumanize the hippie movement as “dropouts” and includes increased criminalization under the Nixon administration (34). It was not until the 1990s when support for legalization began to grow, which mostly overlaps with the NYT data used in the current study.

It has been shown that negative media coverage of substances declined in the 1990s, which coincides with positive public perceptions of marijuana. It is important to note that no causality has been established, as media coverage could be reflective of or driving public perceptions. Stringer and Maggard (61) note that there is also an increase in coverage of medical marijuana during the 1990s, which could be driving this increase in public perception.

Another possible reason for the difference in dehumanization toward people who use marijuana vs. those who use heroin or cocaine is they way these substances are consumed. Both cocaine and heroin can be smoked, snorted, or injected, whereas marijuana is typically smoked or consumed in an edible format. Research has shown that injectable substances are highly stigmatized (62) which can then be operationalized via dehumanization.

Rather than the narrative pushed by the War on Drugs of people with an SUD being cold, incompetent, and subhuman (63), policymakers, providers, and media outlets should ensure that drug policies and everyday healthcare practices counter the components of dehumanization. First, people with an SUD must be reidentified from nonhuman entities like zombies and trash to humans. Second, granting people with an SUD agency requires involving them in paving their road to recovery and recognizing their ability to comprehend potential harms once informed. For instance, the promotion of using drugs only in groups acknowledges the agency of people with an SUD to monitor each other; Good Samaritan laws similarly provide people using drugs immunity and thus the agency to call emergency services for someone else (19). Third, benevolent attitudes rather than negative evaluations of people suffering from SUDs could protect against dehumanization in both clinical and social settings (64). Fourth, blame should primarily be attributed to context and addiction as a disease rather than to the individual to minimize moral disgust. What is tricky is acknowledging agency while shifting blame away from people with SUDs (65). Blaming patients' behavior for their disease plays a role in increasing negative evaluations and moral disgust (66). Disgust evolved to motivate self-monitoring and punish people who threaten others with their disease (67). Disgust has been used to drive the stigmatization of outgroups and limit social interactions when needed most, thus, disgust responses should not always be trusted (68). Some of the disgust mitigation strategies for people with other diseases may apply to people with SUDs. During the AIDS epidemic, for instance, patients fought moral disgust by rejecting self-blame, proving that they do not pose a threat, working together with professionals, and raising awareness about the social effects of disease (67, 69, 70). Finally, research on media depictions of substance users has shown that media outlets are already capable of humanizing PWUS. This split between dehumanizing and humanizing typically falls along racial lines, with urban black and brown people criminalized for injecting heroin while suburban white people who “misuse” prescription drugs are shown in a sympathetic light (71). In sum, the shift away from dehumanization and toward harm reduction can be facilitated by meeting people with SUDs where they are and promoting humanistic treatment strategies to which people with SUDs can more feasibly adhere.

### 6.1 Limitations

The analysis presented here is limited in several ways. First, the selected keywords are by no means exhaustive in terms of variation of substances or substances that are typically dehumanized. For example, we do not consider stimulants, despite the fact that people who use methamphetamines have been referred to as “meth zombies” in popular media and anti-drug ads (34). The keyword *opioid(s)* is perhaps the most obvious missing keyword, given the recent attention the opioid epidemic has received. This term did not gain popularity until after 2010 and, thus, could not be used in a multi-decade analysis. Similarly, keywords such as *xanax*, *percocet*, and *oxycontin* were never frequent enough to analyze, and we were therefore unable to examine prescription opioids. The second limitation is that the keywords do not explicitly refer to substance *users*, with the exception of *addict* and *alcoholic*, but rather the substances themselves. That said, when examining a random selection of articles, we did not find any examples that referred to specific substances such as marijuana and heroin that were not in the context of use. One could use more sophisticated keyword matching (e.g., “cocaine users”) or dependency parsing to identify people who use substances, which may result in a high precision and low recall matching. In the end, we decided on the simpler and more general approach (using substance keywords), since word embeddings and related measures depend on data frequency (72). Third, the baseline of *american* is not ideal, as the New York Times is a U.S. institution and, therefore, this keyword represents an ingroup. Thus, it may be the case that the reported levels of dehumanization toward PWUS are only high compared to this low baseline. That said, the levels of dehumanization reported here for the substance use keywords are similar or higher (e.g., lower distance from vermin) than those reported in Mendelsohn et al. (2) who examined dehumanization toward LGBTQ people, another historically dehumanized group. Fourth, the data was collected using two sampling methods. The data from 1986 to 2015 was collected outside of this manuscript, whereas the data from 2016 to 2020 was collected separately for this study. These collections may have used different sampling strategies and, thus, biased the data from the last 5 years. Finally, we only consider data from a national, highly respected, and liberal-leaning news source. One might expect to see different patterns of dehumanization when using local newspapers, which may report more on drug-related arrests or whose opinion pieces may more accurately reflect the local population. Similarly, right-leaning news sources may also show different patterns of dehumanization.

## Data availability statement

The full dataset is available from the New York Times and can be downloaded using our code: https://osf.io/uya29/. Requests for the 1986–2015 data can be sent to Mendelson et al. (2020) while requests for the 2016–2020 data can be sent to the corresponding author.

## Author contributions

SG: Conceptualization, Data curation, Formal analysis, Methodology, Visualization, Writing—original draft. DH: Writing—original draft. DB: Data curation, Formal analysis, Software, Writing—review & editing. GS: Data curation, Writing—review & editing. BC: Conceptualization, Funding acquisition, Methodology, Supervision, Writing—review & editing.

## Footnotes

^1^https://developer.nytimes.com/docs/archive-product/1/overview.

^2^https://www.crummy.com/software/BeautifulSoup/.

^3^To aid the reader throughout the paper, we italicize keywords in order to distinguish keywords from results, which may be quoted. For example, Table 2 shows that “marijuana” is related to the keyword *heroin* and “heroin” is related to the keyword *marijuana*.

^4^For legibility, we only consider a subset of the keywords.

^5^https://spacy.io.
